# Functionalized Magnetic Nanoparticles for the Detection and Quantitative Analysis of Cell Surface Antigen

**DOI:** 10.1155/2013/349408

**Published:** 2012-12-31

**Authors:** Daryoush Shahbazi-Gahrouei, Mohammad Abdolahi, Sayyed Hamid Zarkesh-Esfahani, Sophie Laurent, Corine Sermeus, Cordula Gruettner

**Affiliations:** ^1^Department of Medical Physics and Medical Engineering, School of Medicine, Isfahan University of Medical Sciences, Hezar Jerib Street, Isfahan, Iran; ^2^Department of Medical Physics, School of Medicine, Bushehr University of Medical Sciences, Bushehr, Iran; ^3^Department of Immunology, Medical School, Isfahan University of Medical Sciences, Hezar Jerib Street, Isfahan, Iran; ^4^Department of General, Organic, and Biomedical Chemistry, NMR and Molecular Imaging Laboratory, University of Mons, 19 Avenue Maistriau, 7000 Mons, Belgium; ^5^Micromod Partikletechnologie GmbH, Rostock, Germany

## Abstract

Cell surface antigens as biomarkers offer tremendous potential for early diagnosis, prognosis, and therapeutic response in a variety of diseases such as cancers. In this research, a simple, rapid, accurate, inexpensive, and easily available in vitro assay based on magnetic nanoparticles and magnetic cell separation principle was applied to identify and quantitatively analyze the cell surface antigen expression in the case of prostate cancer cells. Comparing the capability of the assay with flow cytometry as a gold standard method showed similar results. The results showed that the antigen-specific magnetic cell separation with antibody-coated magnetic nanoparticles has high potential for quantitative cell surface antigen detection and analysis.

## 1. Introduction 

The biomarkers as early warning signs for diseases are physiological and pathological changes in expression level or state, which correlate with the progression in a variety of diseases such as cancers [1, 2]. As the biomarkers show a disease state very specifically and sensitively, they can be used for the early diagnosis, differentiation between disease types with higher accuracy, disease monitoring during and after therapy, and as possible therapeutic targets [3–5]. Among the biomarkers, cell surface antigens play a key role in cellular functions and pathomechanism of diseases in a variety of cancers and since many of them are restrictedly produced against a specific tumor, they can act as ideal biomarkers [6]. It is clear that cancer patients would benefit enormously from a better availability of such effective molecular indicators that help in the development of new diagnostic and therapeutic methods [7, 8].

Although the potential applications of cell surface antigens in cancer diseases appear extraordinarily promising idea, the greatest potential for using this type of biomarkers for cancer lies in improving the technology for cancer cells antigen discovery. So, rapid, simple, accurate, and inexpensive detection methods of the relevant marker are very basic and important. Currently, a wide range of technologies are used for detection and characterization of surface antigens; however, the most widely used method is the analysis of cell surface antigens by flow cytometry [9, 10]. Although the flow cytometry is the gold standard method for accurate and automated measurements of cell surface antigens, this technique is not only expensive and only available in specialized centers but also requires sophisticated equipment and reagents as well as highly trained personnel. Furthermore, in resource-limited countries the access to the technical support and quality assurance programs for flow cytometry is often not readily available [11, 12].

Recently, a new technique has been developed using magnetic nanoparticles coupled to antibodies, as a nonflow cytometric method, which identifies cell surface antigen expression by specific antibody-antigen reaction easier, faster, more efficiently, and at lower cost than the other methods [13]. In addition, the use of magnetic nanoparticles as molecular imaging probes enables noninvasive in vivo studies of antigen expression of diseases in various internal organs [14, 15].

In this work, a rapid and accurate in vitro assay based on magnetic nanoparticles and magnetic cell separation principle was described and developed to discover and quantitatively analyze the cell surface antigen expression of Prostate Specific Membrane Antigen (PSMA). This assay relies on the fact that prostate cancer cells overexpress the PSMA [16, 17].

## 2. Materials and Methods

### 2.1. Materials

Sulfo-SMCC cross-linker (Sulfosuccinimidyl-4-(N-maleimidomethyl) cyclohexane-1-carboxylate), Traut's Reagent (2-iminothiolane), and cysteine were purchased from Sigma Chemical Co. (St. Louis, MO, USA). Nanomag-D-spio nanoparticles in suspension (diameter: 20 nm, surface: CLD-NH_2_, 5 mg/mL; 2.4 mg Fe/mL) were obtained from micromod Partikeltechnologie GmbH (Rostock, Germany). Midi MACS sorting device, LD, and MS high-gradient magnetic field (HGMF) columns were purchased from Miltenyi Biotec GmbH (Gladbach, Germany). PD-10 columns were purchased from GE Healthcare (Piscataway, NJ). The Bradford reagent was purchased from BioRad (Hercules, CA). Amicon centrifugal filters (0.5 mL capacity, 10 kDa MWCO) were purchased from Millipore (Billerica, MA). 

All other chemicals were supplied by Aldrich and used as received. J591 monoclonal antibody was obtained from Professor Neil H. Bander (Cornell University, New York, USA). Cell culture media and fetal bovine serum (FBS) were obtained from GIBCO, Invitrogen Corporation (Carlsbad, CA, USA). Prostate cancer cell lines, DU145 and LNCaP, were purchased from national cell bank of Iran (Pasture Institute, Tehran, Iran) and Cell Lines Service (CLS, Eppelheim, Germany).

### 2.2. Conjugation of J591 Antibody with Nanoparticles

**Figure 1:**
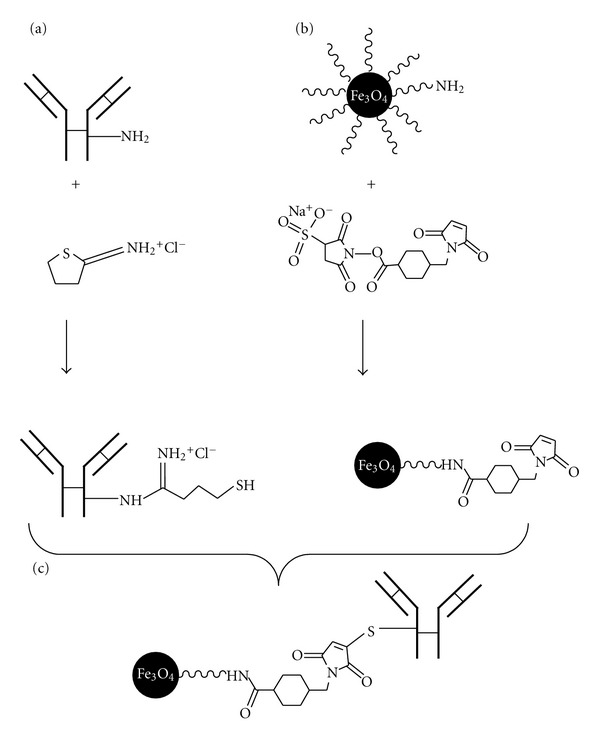
The Scheme of SPIO nanoparticles Conjugation to J591 antibody, (a) functionalization of SPIO-CLD-NH_2_ with Sulfo-SMCC, (b) functionalization of antibody with SH groups using Traut's reagent, and (c) conjugation of thiolated antibody to maleimide functionalized SPIO.

The monoclonal J591 antibody was thiolated and conjugated to maleimide functionalized nanomag-D-spio nanoparticles (Figure 1). Therefore, the sulfo-SMCC cross-linker was first added to nanomag-D-spio particles with CLD-NH_2_ surface to introduce maleimide groups. Specifically, 100 *μ*L of 14.35 *μ*M Sulfo-SMCC solution in DMSO was added to 5 mg of nanoparticles in PBS-EDTA buffer under gentle shaking for 1 h at room temperature. After incubation, the suspension was washed with PBS-EDTA buffer with PD-10 size exclusion columns to remove unreacted sulfo-SMCC. In the next step, primary amines of J591 monoclonal antibodies were modified with 2-iminothiolane to introduce sulfhydryl groups. Typically, 8 *μ*L of 7 mM Traut's reagent solution was added to 400 *μ*L of pure antibody solution in PBS-EDTA buffer (1 mg/mL) and shaken for 1 h at room temperature. To remove unconjugated 2-iminothiolane, the solution was washed three times by 10-kDa cutoff Amicon centrifugal filter units with PBS as an eluent. 

The antibody conjugation to SPIO nanoparticles was achieved by addition of the maleimide functionalized particles to the SH-labeled antibody and incubation under gentle shaking for 3 h at room temperature. Remaining functional groups were blocked by addition of 100 *μ*L of 20 mM freshly prepared cysteine solution. Finally, the antibody-labeled SPIO nanoparticles were purified on magnetic columns (MACS separator). 

### 2.3. Characterization

The hydrodynamic particle size and the width of the particle size distribution (polydispersity index) of nanoparticles were obtained via photon correlation spectroscopy (PCS) using a Malvern Nano Series ZS particle size analyzer (Malvern Instruments, Worcestershire, UK). Samples morphology was observed by transmission electron microscopy (TEM) on a Tecnai 10 TEM (FEI Company, USA) operating at 80 kV. To confirm the feasibility and sensitivity as magnetic cell separation nanoprobe, magnetic properties of synthesized nanoprobe were studied by the use of nuclear magnetic resonance dispersion (NMRD) profiles (Spinmaster FFC 2000, STELAR, Italy), in a field strength range extending from 200 *μ*T to 1.2 T. Additional measurements of relaxation rate (*R*_1,2_) were performed at 20 and 60 MHz on Bruker Minispec system (Bruker, Karlsruhe, Germany). 

The binding of antibody molecules to SPIO nanoparticles and the amount of immobilized antibody were determined by the Bradford assay. Briefly, 40 *μ*L of Coomassie Plus reagent concentrate was added to 160 *μ*L of dispersion of nanoparticles, either mAb-coated or noncoated. After 10 min of incubation, the absorbance was measured at 595 nm using a microplate reader (Stat Fax, Awareness Technologies, USA). The results were compared to a standard curve of BSA solution in the concentration range from 10 *μ*g/mL to 150 *μ*g/mL (Figure 3).

The iron concentrations of the samples were measured by relaxometry measurements at 20 MHz after digestion of samples by microwave oven. This was achieved by mineralization of sample in acidic conditions (0.2 mL sample, 0.6 mL HNO_3_, and 0.3 mL H_2_O_2_) by microwave oven (Milestone MLS-1200, Sorisole, Italy). The millimolar iron concentration was determined from the *R*_1_ relaxation rate of samples, using following equation [18, 19]:

(1) $$\begin{array}{c} \left[\text{Fe}\right]=\frac{\left(R_{1}^{\text{obs}}-R_{1}^{\text{dia}}\right)}{r_{1}\left({\text{s}}^{-1}{\;\text{mM}}^{-1}\right)}, \end{array}$$

where *R*_1_^obs^ is the observed longitudinal proton relaxation rate, *R*_1_^dia^ is the relaxation rate of water protons in the absence of the contrast agent, and *r*_1_ is the longitudinal relaxivity defined as the relaxation rates induced by 1 mmol of iron per liter of solution. *R*_1_^obs^ is the relaxation rate which is measured on the solution containing the sample; *R*_1_^dia^ and *r*_1_ were obtained from a standard curve built by measuring the *R*_1_ of various dilutions of mineralized standard sample of iron (ICP standard, Sigma Aldrich) (Figure 4).

### 2.4. Cell Culture

LNCaP, a PSMA-expressing (PSMA^+^), and DU145, a PSMA negative (PSMA^−^) adherent human prostate cancer cell line, were grown in Dulbecco's Modified Eagle Medium supplemented with 2 mM L-glutamine, 1% NEAA, 1 mM sodium pyruvate, 10% fetal bovine serum, and 1% penicillin/streptomycin (Gibco, Grand Island, NY, USA). For DU145, 1.5 g/L sodium bicarbonate and 0.1 mM nonessential amino acids were used. The cells were cultured in 75 cm^2^ flasks, at 37°C under a humidified 5% CO_2_ atmosphere. For subculture and harvesting the cells, they were washed with PBS followed by treatment with 3 mL TrypLE (Gibco, Grand Island, NY, USA) for 3 min to detach the cells. About 10 mL of culture medium was added to neutralize the TrypLE. The cells were then centrifuged at 3000 RPM for 10 min; the medium was removed and resuspended in complete media and reseeded into new culture flasks.

### 2.5. In Vitro Cytotoxicity

The cytotoxicity of nanomag-D-spio particles and the corresponding J591-antibody-conjugated nanoparticles against LNCaP and DU145 cells was evaluated by using the 3-(4,5-dimethylthiazol-2-yl)-2,5-diphenyltetrazolium bromide (MTT) assay (Sigma-Aldrich) [20]. Exponentially growing LNCaP and DU145 cells were seeded at a density of 2 × 10^4^ cells/well in 96-well plates (Cell Star, Germany). The plates were incubated for 24 h in a humidified incubator with a CO_2_ concentration of 5% to allow adherence of the cells. Once adhered, the cells were incubated with either 0.1 mL of medium containing nanomag-D-spio or SPIO-J591 at iron concentrations ranging from 0.15 to 2.4 mM for 2, 8, and 24 h. The culture medium without any particle was used as the control.

After incubation time 10 *μ*L/well (5 mg/mL), MTT was added and incubation was continued for further 3 h. The medium was carefully removed and the formazan crystals (indicating cell viability) were solubilized by adding 100 *μ*L DMSO (Sigma-Aldrich) per well. The absorbance was determined at 570 nm by the Statfax-microplate reader (Awareness Technology, USA). Experiments were performed in triplicate and cell survival was determined as a percentage of viable cells in comparison with control wells. One-way ANOVA and correlation coefficient between viability and iron concentration were used to determine whether the SPIO nanoparticles caused any significant cytotoxicity.

### 2.6. Fluorescence Microscopy

Fluorescence microscopy was used to qualitatively analyze the cell surface antigen. LNCaP and DU145 cell lines were washed with phosphate buffered saline (PBS), detached by TrypLE, and seeded near confluence (2 × 10^5^ cells/well) on 22 × 22 mm square glass coverslips, which were pretreated with plasminogen in 6-well plates. After attachment, the cells were fixed with 4% formaldehyde solution for 15 min, washed with PBS, then treated with Protein-Free Blocking Buffer (PFBB, Perbio Science, Erembodegem, Belgium) for 1 h at room temperature. The cells were washed with PBS and incubated successively with primary J591 anti-PSMA antibody overnight in the dark at 4°C. After washing with PBS, the cells were incubated with goat anti-mouse FITC monoclonal antibody for an additional 1 h at room temperature in the dark. Cells were then washed, and the slides were mounted using diluted 4′,6-diamidino-2-phenylindole (DAPI) (Vector Labconsult, Brussels, Belgium) solution for 5 min at room temperature and rinsed once with PBS and once with water. The cover slips were mounted onto microscope slides and observed on a confocal microscope (Leica Microsystems, Groot Bijgaarden, Belgium). A semiquantitative analysis of the microscope pictures has been performed by using the ImageJ image analysis software (National Institutes of Health). 

### 2.7. Surface Antigen Expression

In a variety of disease, abnormalities concern not only antigen expression but also its intensity, which may have diagnostic or prognostic significance [21, 22]. Quantitative expression of PSMA on the LNCaP and DU145 cell lines was investigated by flow cytometry, as well as by the new proposed method based on magnetic cell separation. 

To detect cell-surface expression of PSMA by flow cytometry as a gold standard method, indirect immunofluorescence staining was performed. In brief, cells were trypsynized, washed with PBS containing 0.1% fetal bovine serum (FBS), and 10^6^ cells/tube from each cell line were transferred in FACS tubes. The cells were resuspended in 90 *μ*L of washing buffer and were preblocked with FcR Block (human) reagent (Miltenyi) for 10 min at room temperature in the dark. After blocking, primary J591 anti-PSMA antibody (1/150 dilution) was added to each cell tube (one tube of each cell line as a control), incubated for 30 min in the dark at room temperature, and then washed 3 × 5 min using a washing buffer.

 After washing, the cells were resuspended and incubated in goat anti-mouse FITC monoclonal antibody for an additional 30 min at room temperature in the dark. Cells were then washed, resuspended in 0.5 mL of PBS plus 0.1% FBS, and analyzed immediately using a CyAN-ADP flow cytometer (Beckman Coulter). All flow cytometry assessments were repeated at least three times at weekly intervals for each sample.

### 2.8. Magnetic Cell Separation

**Figure 2:**
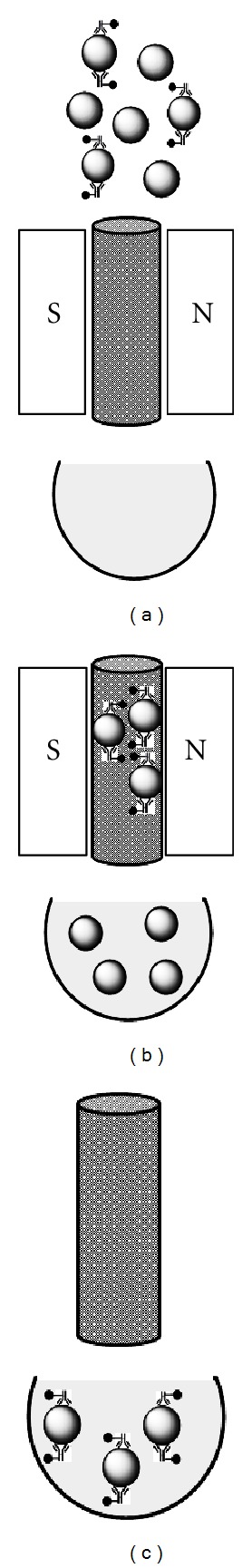
Scheme of the magnetic bead-based cell separation. (a) A mixture of magnetically labeled and nonlabeled cells is applied on a separation column. (b) Specific cell selection using MACS columns. Magnetically labeled cells are retained in the magnetic field of the separation column; unlabeled cells pass through the column as negative fraction. (c) After removal of the column from the magnetic field, the desired cells are eluted as the enriched positive fraction.

Immunomagnetic cell selection as a new method for detection and quantitative expression analysis of PSMA antigens on prostate cancer cells, based on magnetic cell separation technique, has been developed and used (Figure 2).

Human prostate cancer LNCaP cells and DU145 cells were detached and washed three times with PBS. A approximately 1-2×10^6^ cell/tube of each cell type were plated in 15 mL tube and incubated with culture medium containing the synthesized nanoprobe (SPIO-J591) at Fe concentrations of 2 mM. After 2 h incubation at room temperature, cells were washed with PBS three times and resuspended in 1 mL PBS containing 0.1% fetal bovine serum (FBS). The magnetic cell separation was carried out on a midi MACS system. The LS separation column was set in the Midi MACS sorting device, washed twice with 1.0 mL of PBS solution. Then, the cell suspension was added to the separation column, washed three times to obtain the nonmagnetic cells flowing through the sorting column. Finally, the LS separation column was removed from the magnetic field and eluted the double positive cells, and then the PSMA^+^ cells from the MS separation column. The number of PSMA^+^ and PSMA^−^ cells was detected and counted using conventional Trypan blue staining, under an optical microscope. The percentage of PSMA expression on the cell surface was determined using following equation:

(2) The  percentage  of  PSMA  expression =(number  of  PSMA+  cells)(number  of  PSMA+  cells+number  of  PSMA−  cells)  ×100.

## 3. Results and Discussion

### 3.1. Synthesis

**Figure 3:**
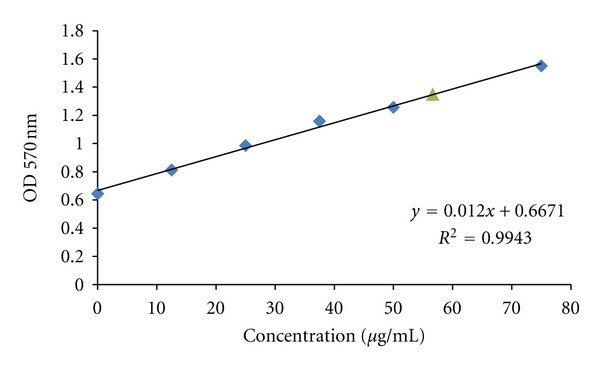
Antibody concentration measurement by Bradford protein assay.

**Figure 4:**
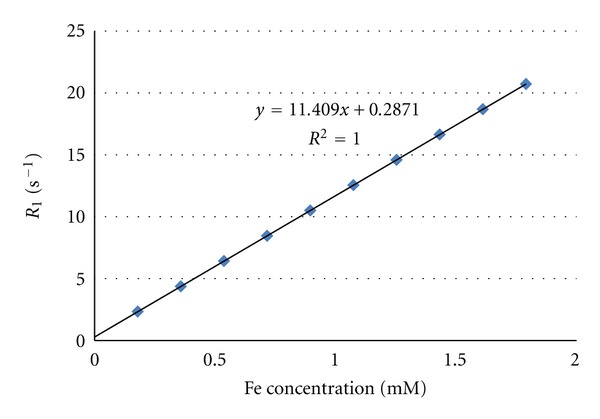
Calibration curve of iron concentration versus relaxation rate at 20 MHz.

The J591 monoclonal antibody was thiolated with Traut's reagent and conjugated to maleimide functionalized SPIO nanoparticles (Figure 1). The feasibility of successfully grafting of antibody molecules to SPIO nanoparticles was confirmed by the Bradford assay as well as the measurements of the hydrodynamic size and shape of SPIO nanoparticles by using PCS and TEM. Analyses by Bradford protein assay and spectrophotometric readings show the amount of immobilized antibody of 56 ± 2 *μ*g Ab/mL of synthesized nanoprobe (Figure 3). Thanks to a standard curve with a commercially available iron standard solution (ICP standard, Sigma Aldrich), the iron concentration of particles was estimated to be 43.88 ± 1.2 and 24.22 ± 0.9 mM for nanomag-D-spio and J591-SPIO, respectively, by MR relaxometry method (Figure 4). 

### 3.2. Characterization

**Figure 5:**
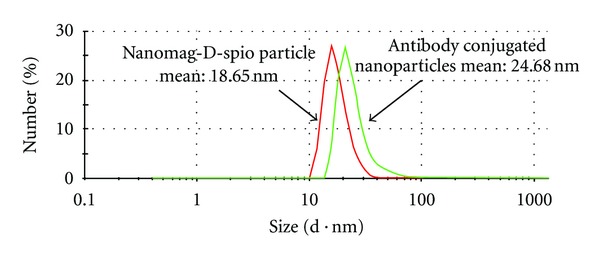
Hydrodynamic diameter of nanomag-D-spio (18.65 nm) and J591-SPIO NPs (24.68 nm).

**Figure 6:**
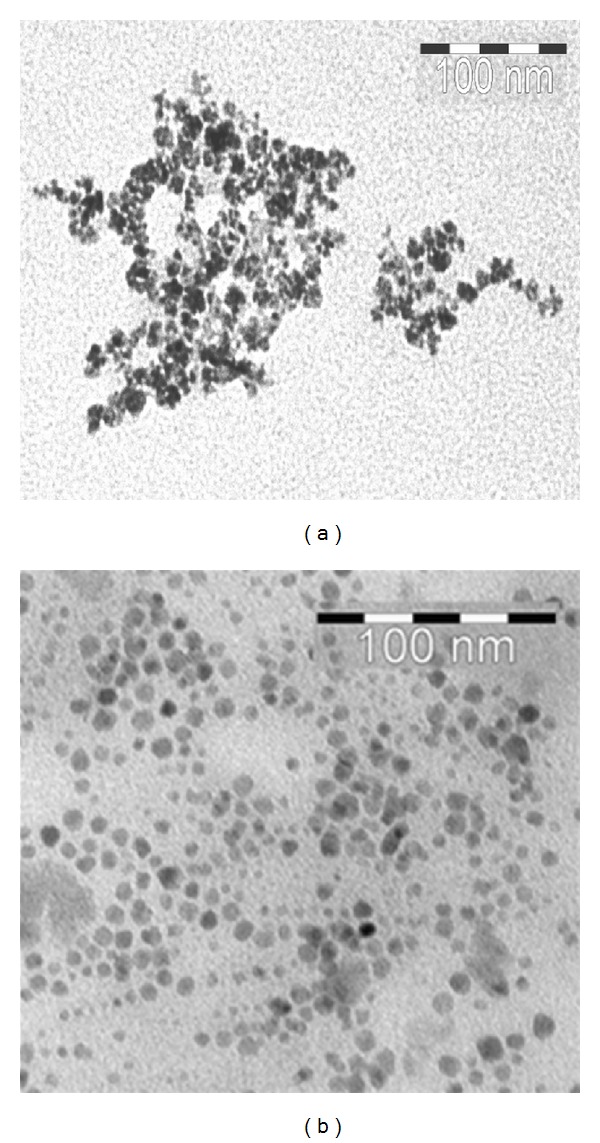
TEM images for plain and antibody conjugated SPIO (a) nanomag-D-spio and (b) SPIO-J591, antibody binding causes a significant reduction of particle agglomeration. The average size of particles estimated from TEM images was about 10–20 nm.

The particle size distribution of SPIO nanoparticles before and after antibody conjugation was determined by PCS (Figure 5). The hydrodynamic particle diameters are determined to be 18.65  ±  0.21 nm for nanomag-D-spio and 24.68 ± 0.22 nm for J591-SPIO. Figures 6(a) and 6(b) show TEM images for the spherical-shaped plain and antibody-conjugated SPIO, respectively. The average particle size calculated from TEM was 10–20 nm for nanomag-D-spio and J591-SPIO. The morphology study of particles from TEM image suggests that antibody molecules conjugated to SPIO nanoparticle reduce the agglomeration of nanomag-D-spio particles.

**Figure 7:**
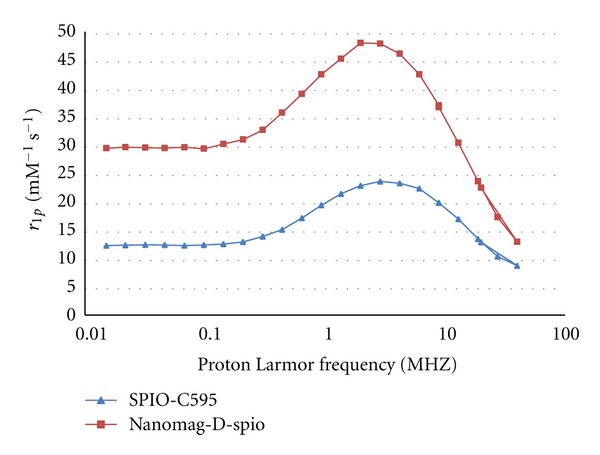
NMRD profiles of nanomag-D-spio and synthesized nanoprobes.

NMRD profiles (Spinmaster FFC2000, STELAR, Italy) were used to examine the magnetic properties of SPIO and J591-SPIO nanoparticles under a magnetic field strength between 200 *μ*T and 1.2 T. The magnetic properties of the SPIO nanoparticles did not change significantly by being conjugated to antibody (Figure 7).

*R*_1_ and *R*_2_ relaxation rate measurements of particles were performed on a Bruker Minispec operating at 20 MHz and 60 MHz. A summary of the longitudinal and transversal relaxivity is provided in Table 1. 

### 3.3. In Vitro Cytotoxicity

**Figure 8:**
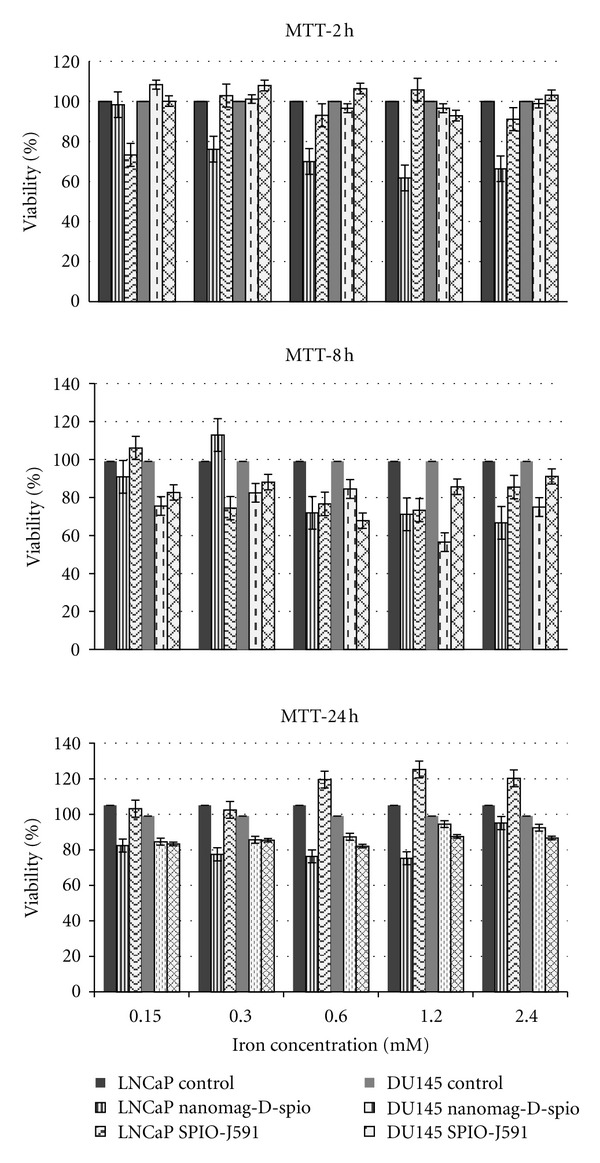
In vitro assessment of cytotoxicity of nanomag-D-spio and SPIO-J591 in LNCaP and DU145 prostate cancer cells by the MTT assay. The cells were incubated with Nanomag-D-SPIO or SPIO-J591 at equivalent iron concentrations ranging from 0.15 to 2.4 mM for 2, 8, and 24 h.

Each nanoprobe for medical application should show minimal toxicity to the targeted cells. The in vitro cytotoxic effect of nanomag-D-spio and the synthesized nanoprobe was assessed using the standard methyl thiazol tetrazolium bromide (MTT) assay, using LNCaP and DU145 cell lines. The results after different incubation times with different iron concentrations for both cell lines show higher than 60% cell viability in relation to the control sample (Figure 8). The statistical analysis by One-way analysis of variance (ANOVA), followed by Duncan's multiple range test, showed statistically significant evidence of nonfunctionalized or functionalized SPIO toxicity to cells (*P* = 0.02). The *P* value between 2 and 8 h, 2–24 h, and 8–24 h incubation of DU145 cell line with SPIO-J591 and nanomag-D-spio was 0.007–0.015, 0.03–0.003, and 0.04–0.36, respectively. In comparison to the LNCaP cell line, these values were 0.17–0.21, 0.15–0.02, and 0.48–0.02. As can be seen, the *P* values are insignificant for most of LNCaP cell line compared to the DU145 cells.

The results of the MTT assay show a moderate negative correlation (*r* = (−0.16)–(−0.71)) between concentration and viability for most of assays after 2 h and 8 h incubation, but after 24 h incubation a positive correlation (*r* = 0.08–0.73) between the concentration and viability was found. 

### 3.4. Cell Surface Antigen Expression

The qualitative and quantitative expression of PSMA on the LNCaP and DU145 cell lines was investigated by fluorescence microscopy, flow cytometry, and proposed method based on magnetic cell separation.

**Figure 9:**
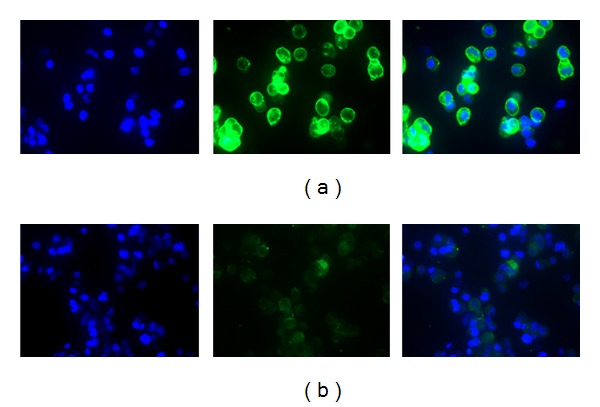
Fluorescent microscopy images (×40 magnification) of LNCaP (a) and DU145 (b), prostate cancer cells after overnight incubation with primary J591 anti-PSMA antibody and additional 1 h incubated with goat anti-mouse FITC monoclonal antibody. Blue colour: DAPI-staining of DNA in the nucleus, green colour: goat anti-mouse FITC bonded to primary J591 anti-PSMA antibody.

The qualitative information on the cell surface antigen expression was obtained by fluorescence microscopy. Figures 9(a) and 9(b) show that the cancer cells were defined by DAPI staining (blue), whilst the green fluorescence represents PSMA^+^ cells. The comparison demonstrates considerably increased fluorescence intensity in the positive LNCaP cells over the negative DU145 cells. 

**Figure 10:**
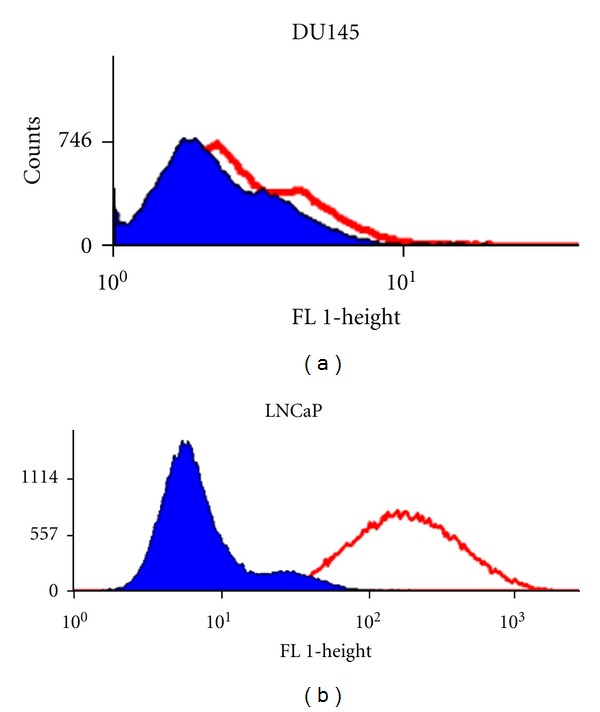
Flow cytometry test shows that DU145 cells lack PSMA expression (a) whereas LNCaP cells express high levels of prostate-specific membrane antigen (PSMA) on their cell surface (b).

Flow cytometry analysis was performed to confirm the availability and quantitative analysis of desired prostate-specific membrane antigen (PSMA) on cell surface. Immunofluorescent staining of LNCaP and DU145 cell lines showed that LNCaP cells express high levels of PSMA on their cell surface (95 ± 1.2%), whereas DU145 cells lack PSMA expression (3 ± 0.2%) (Figure 10). These results are in good agreement with previously published studies [19, 23]. 

Immunomagnetic cell selection was used to determine the ability of functionalized SPIO for detection and quantitative analysis of desired antigen expression on the prostate cancer cells surface. Measurements based on magnetic cell separation method as a proposed immunomagnetic cell selection showed a 94 ± 3.4% and a 6 ± 0.8% expression of PSMA on the surface of LNCaP and DU145 cells, respectively. The statistical analysis between the results of antigen expression by two methods was done using Student's paired *t*-test. Despite statistical significance (*P* Value of 0.02), the actual difference in mean cell surface antigen expression between the flow cytometry and magnetic cell separation method was quite small.

## 4. Conclusions

An increasing number of surface antigens have been characterized in mammalian cell systems during the past decade. It has become obvious that these cell surface structures play an enormous role in early diagnosis, characterization, disease monitoring during and following therapy, and as possible therapeutic targets for various illnesses, especially cancers [24, 25]. However, the difficulty and costs of detection, characterization, and validation of new cell surface antigens has held back rapid development in this field. Hence, the development of a more efficient and inexpensive detection methods of the relevant marker is very basic and important.

Here, the detection and quantitative analyses of PSMA on the prostate cancer cells as an example of a cell surface antigen were described. The iron oxide nanoparticles with anti-PSMA monoclonal antibody (mAb J591) were functionalized to serve as a PSMA-specific molecular probe for in vitro detection and separation of PSMA^+^ prostate cancer cell based on magnetic cell separation technique [26].

Quantitative detection and analysis of desired antigen expression on the cell surface with proposed method was carried out and the results were compared with flow cytometry as a gold standard. Measurements based on the new immunomagnetic cell selection showed an expression of PSMA on the surface of LNCaP and DU145 cells by 94 ± 3.4% and a 6 ± 0.8%, respectively. Whereas using the flow cytometry method, the values of 95 ± 1.2% and 3 ± 0.2%, respectively, have been reported. As the results of both methods are very similar, the magnetic cell separation for the detection and quantitative analysis of the cell surface antigen can be a simple, rapid, accurate, and inexpensive alternative method.

In addition, magnetic separation techniques have several advantages in comparison with traditional techniques for biomarker discovery. It is possible to coat nanoparticles with the ligand of interest, like peptides, aptamers, folic acid, and so forth and use this method to detect and analyze other biomarkers, which is not possible by flow cytometry. Also molecular imaging enables noninvasive in vivo studies of biomarkers of diseases in various internal organs by use of magnetic nanoparticles coupled to targeted reagents [27, 28].

The results obtained from this study prove that use of antibody-coated magnetic beads for isolation of antigen-specific cells is a convenient and simple method for quantitative cell surface antigen detection and analyses.

## Figures and Tables

**Table 1:** Longitudinal and transversal relaxivities (*r*_1_ and *r*_2_) of nanomag-D-spio and synthesized nanoprobe at 20 and 60 MHz at 37°C (Minispec) and the saturation magnetization and size of particles estimated by NMRD profiles data.

Particle	*r*_1_ (s^−1^ mM^−1^)		*r*_2_ (s^−1^ mM^−1^)		*r*_2_/*r*_1_		*M*_sat⁡_ (Am^2^/kg)	*r* (nm)
	20 MHz	60 MHz	20 MHz	60 MHz	20 MHz	60 MHz		
Nanomag-D-spio	21.6	7.6	112	121.8	5.2	16	36.5	8.25
SPIO-J591	14. 9	7.2	83.7	106.5	5.6	14.8	28	8.05
